# Biased and G Protein-Independent Signaling of Chemokine Receptors

**DOI:** 10.3389/fimmu.2014.00277

**Published:** 2014-06-23

**Authors:** Anne Steen, Olav Larsen, Stefanie Thiele, Mette M. Rosenkilde

**Affiliations:** ^1^Laboratory for Molecular Pharmacology, Department of Neuroscience and Pharmacology, Faculty of Health and Medical Sciences, University of Copenhagen, Copenhagen, Denmark

**Keywords:** 7TM-receptor, 7TM structure–function, chemokine system, biased signaling, ligand/receptor/tissue bias, b-arrestin recruitment, G protein coupling, pathway-specific drug development

## Abstract

Biased signaling or functional selectivity occurs when a 7TM-receptor preferentially activates one of several available pathways. It can be divided into three distinct forms: *ligand bias, receptor bias*, and *tissue* or *cell bias*, where it is mediated by different *ligands* (on the same receptor), different *receptors* (with the same ligand), or different *tissues* or *cells* (for the same ligand–receptor pair). Most often biased signaling is differentiated into G protein-dependent and β-arrestin-dependent signaling. Yet, it may also cover signaling differences within these groups. Moreover, it may not be absolute, i.e., full versus no activation. Here we discuss biased signaling in the chemokine system, including the structural basis for biased signaling in chemokine receptors, as well as in class A 7TM receptors in general. This includes overall helical movements and the contributions of micro-switches based on recently published 7TM crystals and molecular dynamics studies. All three forms of biased signaling are abundant in the chemokine system. This challenges our understanding of “classic” redundancy inevitably ascribed to this system, where multiple chemokines bind to the same receptor and where a single chemokine may bind to several receptors – in both cases with the same functional outcome. The ubiquitous biased signaling confers a hitherto unknown specificity to the chemokine system with a complex interaction pattern that is better described as promiscuous with context-defined roles and different functional outcomes in a *ligand*-, *receptor*-, or *cell/tissue*-defined manner. As the low number of successful drug development plans implies, there are great difficulties in targeting chemokine receptors; in particular with regard to receptor antagonists as anti-inflammatory drugs. Un-defined and putative non-selective targeting of the complete cellular signaling system could be the underlying cause of lack of success. Therefore, *biased ligands* could be the solution.

## Chemokine Receptors

**Figure 1:**
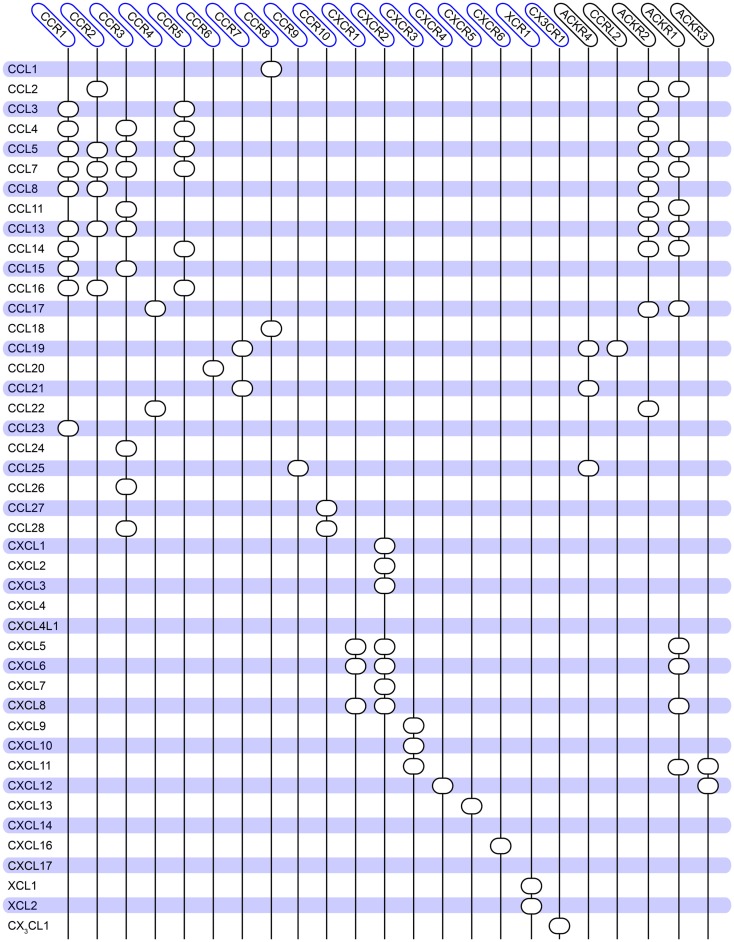
**Overview of the promiscuity of the human chemokine system**. Chemokine ligands are listed vertically, while chemokine receptors are listed horizontally. A circle indicates interaction between the receptor and ligand. No receptors are reported for CCL18, CXCL4, and CXCL14. Atypical chemokine receptors are boxed in black on the right. The diagram is constructed based on (144) and updated to match the database from IUPHAR (2) and the NCBI gene bank^1^.

Chemokine receptors belong to class A 7TM receptors and consist of 350 amino acids on average. Their ligands, the chemokines (8–12 kDa peptides), are divided into two major and two minor groups, depending on the position of two conserved cysteine residues relative to each other. Hence, the two major groups are CCL- (the two cysteines are situated next to each other) and CXCL-chemokines (separated by one amino acid), and the two minor groups are CX_3_CL1 (only one ligand, where there are three amino acids in between the cysteines) and XCLs (two ligands, lacking the first cysteine). The chemokine receptors are grouped according to the ligands they bind, i.e., CCR1-10, CXCR1-6, CX_3_CR1, and XCR1. Presently, there are 18 chemokine receptors and ~40 chemokines acknowledged in the human proteome (1, 2). In addition to these receptors, which all mediate signaling, there are also so-called scavenging receptors, which bind chemokines but have not been shown to mediate intracellular signaling, i.e., ACKR1-4 and CCRL2 (also known as DARC, D6, CXCR7, CCRL1, and CRAM), (2). These are believed to function as decoy receptors to attenuate the chemokine-induced responses. As the large numbers of receptors and ligands indicate, the chemokine system is highly promiscuous. Thus, several chemokines can bind to the same receptor and vice versa (although some receptor–ligand interactions are highly specific and selective, e.g., CCR9–CCL25 and CXCR6–CXCL16). This promiscuous interaction pattern together with the large number of receptors and ligands enables the chemokine system to propagate a great variety of cell functions. An overview of the human chemokine communication network is given in Figure 1.

Chemokine receptors are most often Gα_i/o_-coupled; i.e., they inhibit adenylate cyclase and limit the level of intracellular cAMP and activation of PKA. However, other pertussis toxin-sensitive (i.e., Gα_i_-coupled) effects have been shown to occur in response to activation of chemokine receptors, e.g., phosphorylation of ERK1/2 (part of the MAP kinase cascade) (3–5) and increase in Ca^2+^ flux, most likely through the Gβγ subunit (6), which activates PLC-β. Moreover, it has been shown that Gβγ is important for chemokine-induced leukocyte migration (7, 8) possibly through the action of phosphoinositide-3 kinases (PI3K), thereby stimulating the generation of phosphatidylinositol (3–5)-trisphosphate (PIP_3_) (9, 10).

Chemokines are so named because they are chemotactic cytokines, and thus their primary role is to mediate leukocyte chemotaxis. All chemokines, except CXCL16 and CX_3_CL1, which are integral membrane proteins, are soluble proteins. However, to limit their dissemination, chemokines can bind to negatively charged glycosaminoglycans (GAGs), which are attached to proteins on cell surfaces or the extracellular matrix, forming proteoglycan structures. When secreted by, for example, endothelial cells, chemokines tend to remain concentrated and immobilized at tissue sites, and so do not flow freely in the blood. This immobilization of chemokines is vital for the establishment of a chemokine gradient and thus for the recruitment of leukocytes to endothelial cells lining the blood vessels (11, 12). These chemokine gradients can be made up by *homeostatic chemokines*, which are constitutively produced and coordinate general leukocyte circulation important for immune tolerance as well as maintaining the architecture of secondary lymphoid organs, and *inflammatory chemokines*, which only are produced by activated cells and recruit leukocytes to sites of inflammation. However, some chemokines fall into both categories. In addition to cell migration, other biological functions have been ascribed to chemokine receptors, e.g., angiogenic effects (13, 14), cell-adhesion and cell-extravasation (15, 16), as well as anti-apoptotic signaling (17, 18).

Because chemokines and their receptors are highly important in the immune defense, it is not surprising that they have been attributed roles in several autoimmune and inflammatory diseases. In addition, they are implicated in cancer and viral infections, e.g., HIV. As a consequence, chemokine receptors are amenable drug targets. However, at present only clinical trials in inhibition of HIV infection [the CCR5 antagonist maraviroc (19, 20)] and mobilization of stem cells [the CXCR4 antagonist plerixafor (21, 22)] have successfully led to marketed drugs. In contrast, the pursuit of chemokine-receptor antagonists as anti-inflammatory compounds has been futile, generally because of lack of efficacy in Phase II clinical trials (23, 24). As the number of successful drug development plans implies, there are great difficulties in targeting chemokine receptors. Because of the promiscuity of the chemokine system it is conceivable, that various chemokines binding to the same receptor, and vice versa, induce different responses (25). Drug targeting of a single ligand or receptor could therefore be insufficient to obtain the desired response. On the other hand, antagonizing and thus eliminating physiological responses of exclusive chemokine-receptor pairs could have detrimental effects on for example development, as seen in CXCR4^−/−^ neonatal mice (26, 27). In both cases, un-defined targeting of the complete cellular signaling system could be the underlying cause of lack of success. Therefore, *biased ligands* could be a solution. Because these ligands induce or inhibit selective pathways (see below), this form of treatment could potentially eliminate side effects originating from activation of a variety of cellular signaling pathways. In the following, we will discuss signaling bias from a functional and structural point of view.

## Biased Signaling in Chemokine Receptors

**Figure 2:**
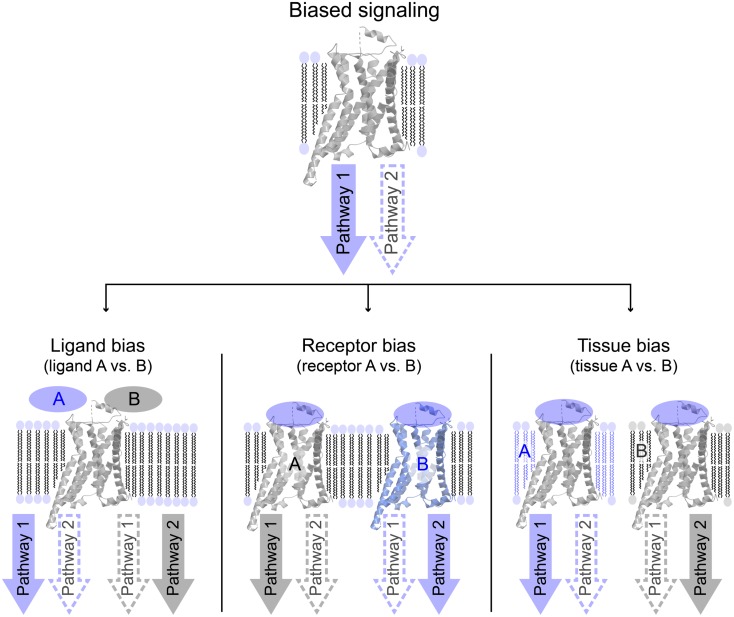
**Overview of different variations of biased signaling**. Biased signaling describes a situation in which a receptor preferentially activates one signaling pathway over another. (Left) Ligand bias is used to differentiate between two ligands acting on the same receptor, where ligand A favorably activates pathway 1, whereas ligand B activates pathway 2. (Center) In receptor bias, the same ligand binds to two different receptors, and activates pathway 1 via receptor A, but pathway 2 through receptor B. (Right) In tissue (or cell) bias, the same ligand: receptor-complex is activated in two different tissues or cell types (or different species), and in tissue A pathway 1 is preferentially activated, whereas pathway 2 is more likely to be activated in tissue B.

*Biased signaling* or *functional selectivity* is a concept, which describes a situation where a 7TM-receptor preferentially activates one of several available cellular signaling pathways. It can be divided into three distinct cases: *ligand bias, receptor bias*, and *tissue* or *cell bias* (Figure 2). Biased signaling is regarded as a relatively new concept, even though a review on the serotonergic system was published as early as 1987, speculating on the potential benefits of selective agonists and antagonists possessing specific effects on a particular receptor-linked effector (28). The concept of biased signaling was introduced by Kenakin in 1995 as “agonist trafficking” (29). Here, it was proposed that agonists have different affinities toward diverse conformational states of the same receptor, which in turn are coupled to individual effector proteins, inducing various signaling pathways. This hypothesis has lately been backed up by several studies (30–33).

Biased signaling can occur both as a result of ligand-induced activation as well as in the absence of a ligand, i.e., via a constitutively active receptor state, as observed for the virus-encoded CXC–chemokine-receptor ECRF3, that signals via Gα_i_ and Gα_q_ in a ligand-dependent manner, but is selective via Gα_i_ when it comes to constitutive activity (34). Most often, biased signaling is differentiated into G protein-dependent and β-arrestin-dependent signaling.

### Ligand biased signaling

*Ligand bias* describes a situation where different ligands bind the same receptor, but induce diverse responses. One example of ligand bias has been proposed for the two endogenous CCR7 ligands, CCL19 and CCL21, which together are involved in the homing of various T cell subpopulations and antigen-presenting dendritic cells (DCs) to the lymph nodes. Here, the T cells are primed by the DCs to allow their antigen-specific activation (35). Although CCL19 and CCL21 bind to the same receptor, they are expressed in slightly different tissues (see below). Moreover, they only share 32% amino acid identity, and importantly, CCL21 has a large C-terminal domain of 37 amino acids that are highly positively charged and capable of binding to glycosaminoglycans (GAGs) and thereby immobilizing the chemokine (36, 37). This is in contrast to CCL19, which does not contain this large C-terminal domain. Furthermore, CCL21 was formerly known as 6Ckine, because it has six cysteine residues in contrast to most other chemokines, which only have four. Due to their differential expression pattern and dissimilar structures, it has been speculated that binding of CCL19 and CCL21 to CCR7 induce distinct cellular responses. Indeed, there is general consensus that whereas both ligands are able to activate G protein-signaling, only CCL19 induces internalization of the receptor (5, 38, 39). Thus, it has been shown that they have the same efficacy in G protein binding (5) and Ca^2+^ flux (4), and also that they show similar efficacy in ERK1/2 phosphorylation (40) (Steen et al., unpublished work). On the other hand, Ricart and coworkers showed that the quantitative chemotaxis of murine DCs against either CCL19 or CCL21 depended on the relative chemokine concentration (41). Thus, at a low chemokine concentration gradient (≤20 nM/mm), CCL19 induced migration with a significantly higher chemotactic index (CI) than CCL21. In contrast, at a chemokine concentration gradient of 200 nM/mm, CCL21 induced migration with a higher CI than CCL19 (41). Furthermore, it has been shown on several occasions that CCL19-mediated β-arrestin recruitment induces receptor internalization whereas CCL21 does not (5, 39, 40). Moreover, Zidar et al. found that in spite of no CCL21-induced desensitization, this chemokine was perfectly able to induce β-arrestin-mediated ERK1/2 phosphorylation. They explained this paradox by a marked GPCR kinase (GRK) preference, i.e., GRK3 activity is unique to CCL19, whereas GRK6 is active in an unbiased manner responding to receptor activation by both ligands (40).

Virus-encoded 7TM receptors often bind a broad spectrum of chemokines, and many cases of ligand bias have been described among these receptors. US28 encoded by human cytomegalovirus binds several human CC-chemokines (CCL1–CCL5), but has particular high affinity for the soluble form of CX_3_CL1 (42). CCL2–5 act as agonists in some pathways and neutral ligands in others, whereas CX_3_CL1 is a partial inverse agonist in most pathways (43–49). Similar phenomena have been observed for the viral CXC-chemokine receptors ECRF3 (from herpesvirus Saimiri) and ORF74 (from human herpesvirus 8) (34, 50–55).

Recently, an article was published addressing the concept of biased signaling in the chemokine system as a possible means for further fine-tuning of the signaling network (56). The authors tested whether different ligands targeting the same receptor displayed bias between G protein-signaling, β-arrestin recruitment, and internalization. They found that at CCR10, CCL28 acted as a G protein-biased agonist, while the other endogenous ligand, CCL27 signaled through both G protein and β-arrestin. Furthermore, from a qualitative bias plot they calculated that at CXCR3, CXCL9 appears to be relatively β-arrestin-biased, while CXCL11 is biased toward internalization. That CXCL11 is the strongest and physiologically most relevant inducer of internalization was already suggested in 2001 (57). A similar phenomenon was described for CXCR2, as CXCL8 was reported to be much more efficient in receptor internalization compared to CXCL7 (58) despite having equally high CXCR2 affinities (59). Later, structural data revealed that the three CXCR3 ligands mediate internalization through different receptor regions. In particular, it was shown that CXCL9- and CXCL10-induced internalization requires serine/threonine residues (putative phosphorylation sites) on the receptor C terminus, whereas CXCL11-induced internalization depends on the third intracellular receptor loop (60). The actual binding sites and overall receptor motifs involved in the binding of the CXCR3 chemokines also vary, as CXCL10 is much more dependent on residues located in the transmembrane helices, i.e., in the deeper receptor regions. Thus, in a study probing the molecular intra-helical requirements for receptor activation in CXCR3 by creating an artificial small molecule binding-site, CXCL11 was “resistant” to most mutants in the helices, whereas CXCL10 binding was severely impaired (61). A similar picture was obtained when the AMD3100 binding-site was successfully transferred from CXCR4 to CXCR3 (62). Finally, a recent study showed that CXCL10 and CXCL11 activate distinct downstream signaling mediators, and lead to a clearly divergent CD4^+^ T cell polarization. Importantly, this bias was demonstrated to be of profound importance for the treatment of experimental autoimmune encephalomyelitis (EAE) in mice, as CXCL11 alone was able to suppress perivascular lesions arising as a result of EAE (63).

The literature also describes incidents of ligand bias in posttranslationally modified chemokines. An example of this is observed for CCL14 and different truncated isoforms binding to the atypical (or scavenging) chemokine-receptor ACKR2. The inactive full-length isoform CCL14 (1–74) binds to ACKR2 but is not internalized and degraded, and does not induce up-regulation of the receptor to the cell surface. This is in stark contrast to the active truncated version CCL14 (9–74), which promotes up-regulation of ACKR2 surface expression and is subsequently degraded, presumably through a G protein-independent, β-arrestin-dependent Rac1–PAK1–LIMK1–cofilin signaling cascade (64, 65). The authors conclude that a proline in position 2 of CCL14 (9–74) is critical for CCL14 degradation and receptor trafficking. In general, chemokine binding to their cognate receptors is postulated to follow a two-step mechanism (66–68). The primary interaction, which provides high-affinity binding to the receptor, is mediated via the chemokine core-domain including the N-loop that follows the conserved cysteine-motif. The second step is an interaction of the ligand N terminus, preceding the first cysteine, with various receptor domains and this is essential for the subsequent receptor activation. In the case of CCL14, the authors propose that a similar interaction occurs between CCL14 and ACKR2. Here, the second step is characterized by the interaction of the second proline of CCL14 (9–74) with the transmembrane bundle of ACKR2, which leads to β-arrestin recruitment and CCL14 degradation. Thus, in the absence of this step, as is the case for the inactive CCL14 (1–74), where the proline is not readily accessible, receptor trafficking is not affected (64). Hence, truncated CCL14 is a ligand biased toward a β-arrestin-dependent activation of the Rac1–PAK1–LIMK1–cofilin signaling pathway. Chemokines undergo other posttranslational modifications, e.g., glycosylation, sulfation, and citrullination [reviewed in Ref. (69)]. It is highly likely that these modifications also can induce biased signaling, enabling different versions of the same chemokine to signal through alternative pathways.

An additional posttranslational event is chemokine dimerization – a phenomenon that also influences chemokine-receptor signaling. For example, at CXCR4 there are distinct signaling patterns associated with monomeric or dimeric CXCL12 that can either promote or inhibit chemotaxis, respectively (70). While monomeric and dimeric CXCL12 activated G protein-dependent signaling (i.e., Ca^2+^ flux and inhibition of forskolin-induced cAMP production) with similar efficacy, the preferential monomeric form of CXCL12 induced a greater response in β-arrestin recruitment and chemotaxis. Moreover, whereas activation of CXCR4 by monomeric CXCL12 resulted in a slower increase in ERK1/2 phosphorylation, dimeric CXCL12 induced a rapid and transient phosphorylation (70).

As CXCR4 is one of the primary coreceptors for HIV infection, many compounds targeting this receptor have been synthesized. One example is the class of pepducin molecules, which are lipid-modified peptides derived from the amino acid sequences of one of the three intracellular loops of a target 7TM receptor (71). One of these pepducins, ATI-2341, has been shown to act as a biased agonist on CXCR4, being able to induce β-arrestin coupling but not Gα_i_ signaling in contrast to CXCL12 (72). Thus, also synthesized compounds can exhibit biased properties.

### Receptor biased signaling

*Receptor bias* refers to the case where the same ligand induces different responses on different receptors. For example, the chemokine CXCL12 has been shown to bind to both CXCR4 and ACKR3. CXCR4 was originally thought to be the sole receptor for CXCL12 in the otherwise promiscuous chemokine system. As mentioned above, CXCL12 induces activation of both Gα_i_ (73) and β-arrestin via CXCR4 (74, 75). In 2005, it was discovered that CXCL12 also bind to ACKR3 (76), but this chemokine receptor was thought to act as a scavenger, sequestering CXCL12 inside the cell, as no immediate G protein-signaling was observed for this ligand:receptor interaction (77, 78). However, it was soon discovered that CXCL12 is able to activate β-arrestin-mediated *signaling* in ACKR3 (79), and this distinguishable activation on ACKR3 with respect to CXCR4 makes it the first case of endogenous receptor biased signaling.

Along those lines, atypical chemokine receptors have been described as being able to bind a variety of chemokines but without eliciting Gα_i_ signaling. They were originally thought to regulate the amount of free chemokine available to bind to chemokine receptors (i.e., “scavenge”) and thus dampen an immune response. However, incidents have since been described – as for ACKR2 – where they might be able to induce G protein-independent intracellular signaling. ACKR2 binds most inflammatory CC-chemokine agonists of CCR1–5 (80) and no G protein-signaling has been recorded upon binding of any of them. Although β-arrestin-dependent signaling has not been described for all the chemokine ligands, it is conceivable that this form of biased signaling occurs in other incidents than the described ACKR2–CCL14 interaction (see above).

A different side to chemokine-receptor bias is the discovery that several chemokines, which have been dubbed natural *agonists* for some chemokine receptors, act as natural *antagonists* for other chemokine receptors. One example is the chemokines targeting CXCR3, i.e., CXCL9, -10, and -11, which originally were shown to be selective agonists for this receptor. However, it has been shown that the three ligands are able to displace the binding of CCL11 on CCR3-expressing cells (81). Moreover, they are all able to inhibit CCL11-induced CCR3^+^-cell migration and (cytoplasmic) [Ca^2+^*_i_*] changes with no intrinsic activity, and in addition, neither of the CXCR3 agonists induces CCR3 internalization, and thus lack agonistic effects but act as full antagonists (81). In another setting, it has been shown that CXCL11 also acts as a CCR5 antagonist that inhibits the binding of CCL3 to CCR5-transfected cells and reduces cell migration in response to CCL5 and CCL4, the latter being a selective agonist for CCR5. In addition to the abovementioned examples, a range of other chemokines have been described as natural antagonists, e.g., CCL26 on CCR1 and -5 (82), CCL18 on CCR3 (83), CCL7 on CCR5 (84), CCL4 on CCR1 (85), and CCL11 and CCL26 on CCR2 (86, 87). For CCL11, the action is even more complex, as it also activates certain pathways in CCR2 (88). It remains to be determined if these antagonistic (and complex agonistic) properties can be explained by the lack of (or altered) secondary step(s) in the two-step activation mechanism for chemokine receptors.

Viral chemokine receptors display biased signaling relative to their human counterparts responding to the same ligand. For example, CX_3_CL1 is an inverse agonist at US28 via Gα_q_, although it is a full agonist for the endogenous receptor CX_3_CR1 via Gα_i_ (46, 89). Likewise, CXCL6, -10, and -12 are inverse agonists for ORF74–HHV8 via Gα_q_ (52–54) but full agonists on their cognate human receptors via Gα_i_ (73, 90–92). Finally, receptor bias also occurs between viral receptors as observed for ORF74–HHV8 and ECRF3–HVS, where various CXCLs bind with different efficacies and initiate different pathways in these two receptors (34, 50, 54, 55, 93, 94).

### Tissue bias

*Tissue bias* (also referred to as *system or cell bias*) covers the phenomenon in which a ligand for a given receptor activates different pathways in a tissue/cell-specific manner – or in a species-specific manner. The phenomenon has been described in non-chemokine class A 7TM receptors, e.g., in the β-adrenergic receptors, where Kenakin and coworkers observed a system bias (95, 96). Although there has not been much focus on tissue bias *in vivo*, it is very likely that efficiency of coupling to various cellular pathways is tailored to the needs of the cell. For example, the two endogenous ligands for CCR7, CCL21, and CCL19, show variation in their expression pattern (described above); i.e., CCL19 is expressed by mature DCs, whereas CCL21 is expressed in afferent lymphatic vessels, and they are both present in the lumen of high endothelial venules (HEVs) and on stromal cells of the lymph nodes (97–99). Their differential expression pattern and very diverse structures indicate individual roles for the two chemokines. Indeed, different articles disclosing the role of CCL19 in chemotaxis show that the efficacy is cell-type-dependent; CCL19 is able to induce chemotaxis of DCs (41), whereas CCR7 expressing T lymphocytes are unable to migrate to secondary lymphoid tissue in response to this chemokine (100). Thus, CCL19 has a differing role in inducing chemotaxis depending on cell type, and these results might be extrapolated to other chemokines.

Another example of tissue bias is seen for the CXCR4-specific small molecule ligand AMD3100 that was originally designed as an antagonist hindering the ability of HIV to interact with CXCR4 and thus infect the cell. The binding mode of AMD3100 in CXCR4 has been thoroughly characterized; it not only inhibits the binding of HIV, but also that of the CXCR4-specific antibody 12G5 and CXCL12, and thereby also inhibits the CXCL12-induced Gα_i_ activation and intracellular Ca^2+^ release (62, 101–105). These results were achieved by using SUP-T1 cells or COS-7 cells. On the other hand, AMD3100 has been reported to act as a *partial* agonist on WT CXCR4 in a Ca^2+^ release assay, and with even higher efficacy on a constitutively active CXCR4 mutant in THP-1 cells (106). Thus, as well as being a biologically integrated beneficial phenomenon for tissues *in vivo* (like CCR7 and CCL19/CCL21), tissue bias can be an obstacle when trying to determine the efficacy of a ligand/receptor *in vitro* or *ex vivo*. Hence, to be fully able to determine the biased nature of a ligand or a receptor, comparison between the ligand/receptor-complex in the same tissue or cell type is vital; lessons that are important in particular for drug discovery within 7TM receptors (see below).

## Structural Basis for Biased Signaling in Chemokine Receptors, and in Class A 7TM Receptors in General

As other membrane proteins, 7TM receptors are highly dynamic and exist in several functionally distinct conformations (107). Yet, it is generally acknowledged that the activation of all class A 7TM-receptor subclasses involves the same overall helical movements (108–110) and that specific domains, so-called micro-switches, regulate these movements (111, 112). In order to develop drugs targeting a limited number of the signaling pathways of a given receptor (biased drugs), it is vital to understand, which residues are important for ligand binding and subsequent receptor interaction with different intracellular effector molecules. Hence, much attention has been focused on disclosing the conformations of 7TM receptors interacting with either the G proteins or β-arrestins, both by developing “static” pictures of active and inactive receptor conformations by X-ray crystallography and nuclear magnetic resonance (NMR) spectroscopy, but also in obtaining a more dynamic view of the activation process by employing structure–function studies. The only chemokine-receptor structures that have been published are of CXCR4 (113), CCR5 (114), and CXCR1 (115) and none of them have been in complex with an effector molecule. Hence, these structures do not describe, which receptor conformations are able to bind to either G protein or β-arrestin. However, the chemokine-receptor structures share an overall three-dimensional shape with other class A 7TM receptors and therefore, it is conceivable that results from these structures can be transferred to the chemokine receptors.

### Interactions with G protein

**Figure 3:**
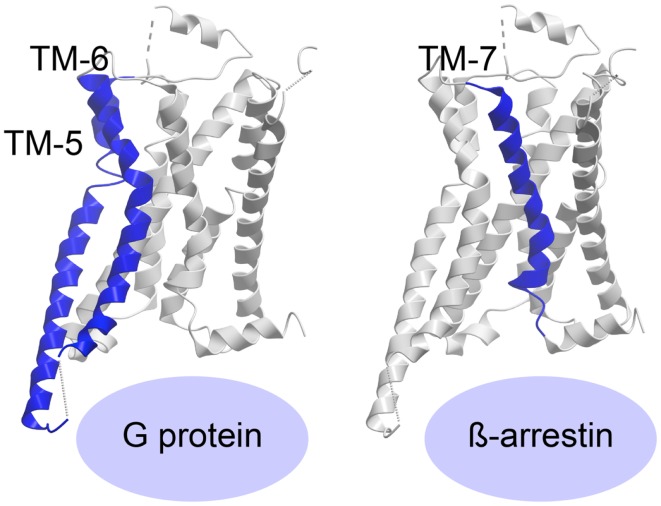
**Activation of a 7TM receptor**. The active β_2_-adrenergic receptor with TM-5 and -6 or -7 highlighted in blue according to their importance for receptor interaction with G protein or β-arrestin, respectively. The structures are visualized with Molsoft Browser Pro©.

The recently published crystal structure of the β_2_-adrenergic receptor in complex with agonist and G protein was the first truly active G protein-coupled 7TM receptor to be disclosed (116). As suggested by Rasmussen et al., engagement of the G protein by the receptor leads to dramatic changes in the Gα subunit, which in turn forces displacement and stabilizes the β_2_-adrenergic helices TM-5 and -6, curving TM-6 extensively outward (Figure 3) (116, 117). The β_2_-adrenergic receptor/agonist/G protein complex also reveals that the interface between the G protein and the receptor involves ICL-2 in addition to TM-5 and -6. Importantly, the structure shows that there is no interaction between the receptor and the Gβγ subunit and furthermore, that there is no interaction between the G protein and TM-7 and helix-8 of the receptor.

### Receptors in complex with ligands activating β-arrestin

Most of the 7TM-receptor crystal structures published so far have focused on G protein activation, as the ligands employed have been known as G protein agonists. Furthermore, most crystals have been obtained with truncated and/or otherwise modified receptors. Although β-arrestin has been crystallized in complex with the phosphorylated C-terminal tail of vasopressin (118), no structure of β-arrestin (or the protein kinase A or C, PKA or PKC, or the G protein-coupled receptor kinases, GRKs, responsible for C-terminal receptor phosphorylation) in complex with an entire 7TM-receptor has yet been crystallized. As such, the required ligand-induced receptor conformation priming β-arrestin is difficult to delineate. However, recently published structures of the β_1_-adrenergic receptor (119) and the serotonin 5-HT_2B_ receptor (120) have been in complex with agonists, which preferentially initiate β-arrestin-signaling. The structure of ergotamine (ERG) in complex with either 5-HT_2B_ or 5-HT_1B_ was compared, and ERG acts as a β-arrestin-biased agonist on 5-HT_2B_ but *unbiased agonist* (equal potency and efficacy in G protein- and β-arrestin-signaling) on 5-HT_1B_. This enabled the authors to pinpoint molecular characteristics in the β-arrestin-favorable receptor conformation. By comparing the two structures with the β_2_-adrenergic receptor/agonist/G protein structure, they were able to deduce, which domains of the two serotonin receptors adopted an active-like conformation. They discovered large changes both in overall helical rearrangements as well as in micro-switches [amino acid sequence motifs that are highly conserved in the class A 7TM-receptor family, i.e., PIF (Pro–Ile–Phe) in TM-3, -5, and -6, D/ERY (Asp/Glu–Arg–Tyr) in TM-3, and NPxxY (Asn–Pro–x–x–Tyr) in TM-7]. For example, in the β_2_-adrenergic receptor:agonist:G protein complex, the PIF motif was seen to significantly change conformation from the inactive structure and while all these changes also occurred in 5-HT_1B_, only the active-like conformation of ProV:16/5.50^2^ and IleIII:16/3.40, but not PheVI:09/6.44, was observed in 5-HT_2B_. In addition, the ionic lock formed by the D/ERY motif in inactive 5-HT receptor structures was broken in 5-HT_1B_ but not in 5-HT_2B_. In contrast, both serotonin receptors (5-HT_1B_ and 5-HT_2B_) displayed active-state conformations of the conserved NPxxY motif in TM-7, but with more pronounced activation features in the 5-HT_2B_ receptor. Importantly, overall helical changes indicated that whereas the 5-HT_2B_/ERG structure showed less pronounced active-like changes in TM-6, TM-7 appears to be in a more active conformation than in the 5-HT_1B_/ERG structure. Accordingly, the authors deduced that while TM-6 is extremely important for G protein binding [in agreement with the active structures of the β_2_-adrenergic receptor (116, 121)], TM-7 is highly involved in β-arrestin coupling (Figure 3). Along those lines, it was observed that the β-arrestin-biased β_1_-adrenergic receptor agonists, carvedilol and bucindolol compared to unbiased β_1_-adrenergic agonists such as isoprenaline, interacted with additional residues in TM-7 and ECL-2 (119, 122).

The molecular mechanisms that occur in G protein and/or beta-arrestin recruitment are further substantiated in a recent publication concerning the delta-opioid receptor (123). Here the authors addressed the role of a transmembrane asparagine residue (N131 in position III:11/3.35). When substituting this with alanine (N131A), the receptor lost the ability to signal through Gα_i_, but constitutively (124, 125) recruited β-arrestin. Thus, this mutation generated a receptor with biased signaling as compared to WT delta-opioid receptor; a phenomenon that at the molecular level was coupled to loss of the sodium ion coordination in the mutated receptor.

In addition to the published structures, dynamic studies, such as structure–function studies or spin labeling, have indicated, which receptor domains are involved in G protein and β-arrestin binding. For example, a study employing site-specific ^19^F-NMR labels in the β_2_-adrenergic receptor showed that the cytoplasmic ends of TM-6 and TM-7 adopt two conformational states depending on whether the receptor is in complex with β-arrestin-biased ligands or non-biased ligands. Hence, unbiased agonists primarily induce shifts toward the active state of TM-6, while β-arrestin-biased agonists predominately impact the conformational states of TM-7 (31). In the chemokine world, mutational studies have primarily been used to gain knowledge about domains relevant for activation. A mutagenesis study in CCR5 showed that mutation of R126 in the DRY motif (in the bottom of TM-3) to a neutral amino acid abolished CCR5-mediated G protein activation, but induced a higher level of receptor phosphorylation and β-arrestin coupling, thus producing a β-arrestin-biased receptor (126). Lack of G protein activation, while retaining β-arrestin coupling, resembles the signaling pattern of the atypical chemokine receptor as discussed above. Interestingly, these receptors often differ from conventional chemokine receptors in the highly conserved DRYLAIV motif. However, it has been shown on several occasions that “correction” of the variations in the DRYLAIV motif in the atypical chemokine receptors does not induce G protein-signaling through these receptors. For example, replacing the ACKR3 ICL-2 with that of CXCR4 – and thus exchanging DRYLSIT with DRYLAIV – did not enable ACKR3 to activate the G protein-dependent signaling pathways (127). Moreover, modifications of the DRYLAIV motif are also seen in the chemokine receptors XCR1 and CXCR6, which have been shown to signal through pertussis toxin-sensitive Gα_i_ protein (128, 129). Thus, lack of or a mutated DRYLAIV motif does not represent a reliable indicator for the lack of G protein coupling, and indeed, the chemokine receptors that lack the conserved arginine in the DRY motif [like the CXCL receptor encoded by equine herpesvirus 2 (ORF74-EHV2)] also signal through G proteins (130, 131). Similarly, a recent study showed that this conserved arginine indeed is dispensable for G protein-signaling in the β_2_-adrenergic receptor (132).

## Helical Movements and Micro-Switches Involved in CCR5 Activation of Different Signaling Pathways

**Figure 4:**
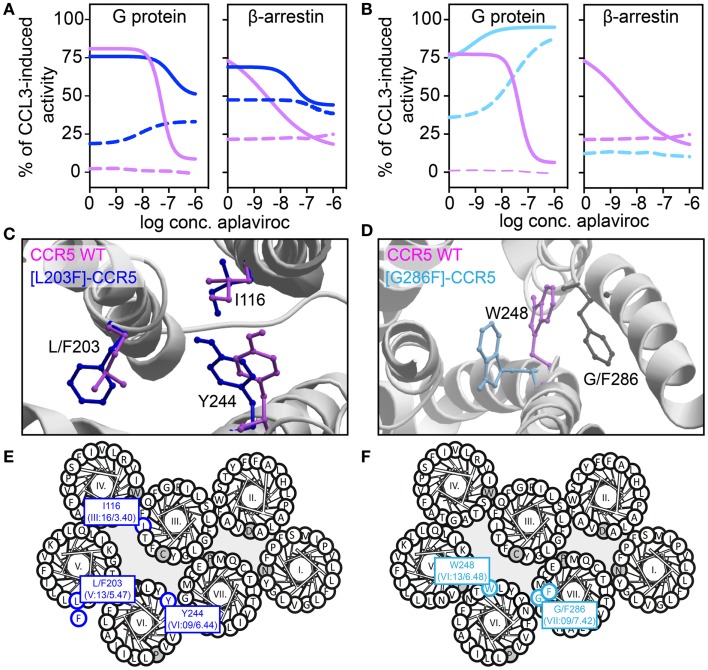
**Biased activity in CCR5**. **(A,B)** Dose–response curves showing the aplaviroc-mediated activation and inhibition of CCR5 WT (purple), [L203F]-CCR5 (dark blue), and [G286F]-CCR5 (light blue) in G protein and β-arrestin-signaling. Aplaviroc was tested with (full lines) or without (dotted lines) the endogenous ligand CCL3. **(C,D)** Computational models of the WT and mutant receptors depicting the difference in side chain conformations of important amino acids. **(E,F)** Helical wheel diagrams showing the positions of relevant amino acids.

We have recently described transmembrane residues of importance for G protein-signaling and β-arrestin binding in CCR5 (133, 134). In brief, insertion of a steric hindrance mutation in either the center of TM-5 (L203F, in position V:13/5.47) or in TM-7 (G286F, in position VII:09/7.42) resulted in constitutive Gα_i_ activity and increased the affinities of endogenous chemokines. A computational model of [G286F]-CCR5 revealed that the conserved tryptophan in TM-6 (W248 in CCR5, part of the CWxP-motif) changed orientation away from TM-7 compared to WT. The essential role of W248 in CCR5 activation was supported by complete inactivity of [W248A]-CCR5. Thus, the altered positioning of W248 – induced by G286F – led to a constraint of a more agonist-prone nature. On the other hand, a conformational change of a conserved hydrophobic residue in position III:16/3.40 in TM-3 (I116 in CCR5) was observed in an *in silico* model of [L203F]-CCR5. Furthermore, a sliding movement of Y244 in TM-6 (position VI:09/6.44) toward TM-5 was observed. *In vitro*, [I116A]-CCR5 displayed the same level of constitutive activity as [L203F]-CCR5, whereas [Y244A]-CCR5 could not be activated. This could indicate that I116 serves a gating function for Y244 movement toward L203, which is important for G protein-signaling. A similar role of these two residues was previously described for other class A 7TM receptors [i.e., the ghrelin receptor, GPR119, NK-1, and the β_2_-adrenergic receptor (135)]. Both ligand-dependent and -independent β-arrestin recruitment was eliminated in [G286F]- and [W248A]-CCR5, indicating biased signaling. In contrast, [I116A]- and [L203F]-CCR5 were able to recruit β-arrestin both in the presence and absence of agonist. Thus, tampering with the interplay between TM-3 and -5 increases the level of both Gα_i_ signaling and β-arrestin recruitment, while manipulation of the interface between TM-6 and -7 increases G protein-signaling, reduces β-arrestin recruitment and hence, induces biased signaling (Figure 4).

In the abovementioned CCR5-studies, characterization of constitutive activity and activation in response to small molecule ligands on signaling revealed that the CCR5 antagonist aplaviroc underwent an *efficacy switch*, i.e., from being antagonist in G protein-signaling on WT to an agonist on [L203F]- and [G286F]-CCR5. However, as this switch was not observed in β-arrestin recruitment aplaviroc acted as a biased agonist on these mutants (Figure 4), indicating that receptor domains can regulate coupling to different pathways independently as also observed in the delta-opioid receptor. Using compounds like aplaviroc for structure–function experiments provides valuable tools to gain knowledge of the specific receptor domains and ligand structures that induce biased signaling.

## Conclusion

The experimental results discussed in this review illustrate that biased signaling is a complex phenomenon and that it exists to a large degree in the chemokine system. Biased signaling can occur in different forms; signaling can vary (1) with the ligand, (2) with the receptor, and (3) with the tissue or cell type (or species). All three factors should be taken into consideration when designing, developing, and testing new compounds targeting a component in the chemokine system as well as in the overall 7TM-receptor family. For example, when testing a potential pharmaceutical product *in vitro*, it is not enough to assess its properties against only one endogenous agonist, nor is it sufficient to measure the activity through one signaling pathway. Furthermore, if the target of interest is expressed in diverse tissues it is necessary to ensure that the observed response is identical in other tissues or cell types. In many cases bias is not absolute, i.e., full activation of one pathway and no activation of another. Often, there is activation in all pathways tested and varying efficacies and/or potencies can be defined as a bias. This variation can of course be caused by a natural preference, but it can also be due to cell type (if the different outputs are measured in different cell types) or sensitivity of the assay type. Therefore, it is important to compare efficacies and/or potencies within the assay and cellular systems and thus obtain a bias output that is relative.

The chemokine system is an obvious target for biased ligands due to the high degree of redundancy where multiple ligands bind to the same receptor and therefore potentially elicit an array of intracellular signaling patterns. Thus, designing a selective compound for one signaling pathway would in theory lower the side effects that would arise when inhibiting/activating all signaling pathways. The redundancy of the chemokine system is however impedimental when designing chemokine-targeting drugs, as (1) other ligand–receptor combinations can have a compensating function, and (2) as drugs may be selective for one, but not all chemokines for a given receptor [e.g., in CCR1, where small molecule ligands acted as positive allosteric modulators with respect to CCL3, but at the same time competed against CCL5 (136)]. Furthermore, small drug-like substances may act differently on homologous chemokine receptors, as observed in CCR1 and CCR5, where a chloro-terpyridine-based compound acted as agonist with no allosteric properties on CCR1, but with the opposite phenotype on CCR5 (137). This compound was developed from the bipyridine and phenanthroline-based non-selective CCR1, -5, and -8 ago-allosteric compounds (137, 138).

Chemokines mainly interact with extracellular receptor regions (68), and therefore the two conformationally constraining disulfide bridges have a major impact on chemokine binding (113, 115, 139). Yet, the roles of these bridges vary – even between homologous chemokine receptors like CCR1 and CCR5 (139) and thereby contribute to the explanation for why small drug-like substances act differently on (1) different chemokines for the same receptor (136) or (2) different chemokine receptors (137, 138). These pharmacodynamic challenges combined with the redundancy of the chemokine system may be central for the lack of success when it comes to the development of anti-inflammatory compounds targeting chemokine receptors. *Biased ligands* could be a solution with a directed action on a specific chemokine-receptor domain, and thereby preferential targeting of a single intracellular pathway. This more focused action could potentially eliminate lack of drug efficiency caused by redundancy and chemokine cross-talk, and also eliminate side effects stemming from unspecific activation of a variety of cellular signaling pathways.

Interestingly, biased ligands have been developed in other receptor systems, especially in the cardiovascular system. For example, carvedilol is an unspecific β-blocker with especially good clinical efficacy. It has been shown to be an inverse agonist in Gα_s_-signaling while it stimulates phosphorylation of the C-terminal tail and concomitant β-arrestin recruitment and internalization as well as ERK1/2 activation in HEK-293 cells (140). Furthermore, carvedilol has been shown to transactivate epidermal growth factor receptor (EGFR, a receptor tyrosine kinase) and mediate ERK1/2 activation in a G protein-independent fashion (141). The transactivation of EGFR has been shown to be cardioprotective (142), whereas chronic β-adrenergic receptor coupling to Gα_s_ is thought to be cardiotoxic (143) and this might explain the unique clinical profile of carvedilol.

In conclusion, deciphering the mechanisms behind biased signaling, including which receptor domains and ligand compositions are responsible for activation of one effector molecule over another is crucial when designing and developing potent and specific 7TM-receptor pharmacotherapeutics. In the chemokine system, signaling bias is abundant in all three forms: ligand, receptor, and tissue (cell/species) bias. With this continuous uncovering of signaling bias in the chemokine system, it becomes clear that the apparent redundancy, which seems inevitably coupled with this system, may be a superficial phenomenon. Instead, the concept of promiscuity may be more correct, and it would be prudent to consider that promiscuous chemokines and their receptors likely have context-defined and separate roles, including the possibility for different functional outcomes of seemingly redundant interactions.

## Conflict of Interest Statement

The authors declare that the research was conducted in the absence of any commercial or financial relationships that could be construed as a potential conflict of interest.

## Footnotes

^1^http://www.ncbi.nlm.nih.gov/gene/

^2^Amino acid positions are given according to the Baldwin-Schwartz-, followed by the Ballesteros-Weinstein nomenclature (124, 125).

## References

[1] Murphy PM Baggiolini M Charo IF Hebert CA Horuk R Matsushima K International union of pharmacology. XXII. Nomenclature for chemokine receptors. Pharmacol Rev (2000) 52(1):145–76 10699158

[2] Bachelerie F Ben-Baruch A Burkhardt AM Combadiere C Farber JM Graham GJ International Union of Pharmacology. LXXXIX. Update on the extended family of chemokine receptors and introducing a new nomenclature for atypical chemokine receptors. Pharmacol Rev (2014) 66(1):1–79 10.1124/pr.113.007724 24218476 PMC3880466

[3] Ganju RK Brubaker SA Meyer J Dutt P Yang Y Qin S The alpha-chemokine, stromal cell-derived factor-1alpha, binds to the transmembrane G-protein-coupled CXCR-4 receptor and activates multiple signal transduction pathways. J Biol Chem (1998) 273(36):23169–75 10.1074/jbc.273.36.23169 9722546

[4] Yoshida H Nagira M Kitaura M Imagawa N Imai T Yoshie O Secondary lymphoid-tissue chemokine is a functional ligand for the CC chemokine receptor CCR7. J Biol Chem (1998) 273:7118–22 10.1074/jbc.273.12.7118 9507024

[5] Kohout TA Nicholas SL Perry SJ Reinhart G Junger S Struthers RS Differential desensitization, receptor phosphorylation, beta-arrestin recruitment, and ERK1/2 activation by the two endogenous ligands for the CC chemokine receptor 7. J Biol Chem (2004) 279(22):23214–22 10.1074/jbc.M402125200 15054093

[6] Murdoch C Finn A Chemokine receptors and their role in inflammation and infectious diseases. Blood (2000) 95(10):3032–43 10807766

[7] Arai H Tsou CL Charo IF Chemotaxis in a lymphocyte cell line transfected with C-C chemokine receptor 2B: evidence that directed migration is mediated by betagamma dimers released by activation of Galphai-coupled receptors. Proc Natl Acad Sci U S A (1997) 94(26):14495–9 10.1073/pnas.94.26.14495 9405641 PMC25033

[8] Neptune ER Bourne HR Receptors induce chemotaxis by releasing the betagamma subunit of Gi, not by activating Gq or Gs. Proc Natl Acad Sci U S A (1997) 94(26):14489–94 10.1073/pnas.94.26.14489 9405640 PMC25031

[9] Hirsch E Katanaev VL Garlanda C Azzolino O Pirola L Silengo L Central role for G protein-coupled phosphoinositide 3-kinase gamma in inflammation. Science (2000) 287(5455):1049–53 10.1126/science.287.5455.1049 10669418

[10] Rot A Von Andrian UH Chemokines in innate and adaptive host defense: basic chemokinese grammar for immune cells. Annu Rev Immunol (2004) 22:891–928 10.1146/annurev.immunol.22.012703.104543 15032599

[11] Rot A Endothelial cell binding of NAP-1/IL-8: role in neutrophil emigration. Immunol Today (1992) 13(8):291–4 10.1016/0167-5699(92)90039-A 1510812

[12] Tanaka Y Adams DH Shaw S Proteoglycans on endothelial cells present adhesion-inducing cytokines to leukocytes. Immunol Today (1993) 14(3):111–5 10.1016/0167-5699(93)90209-4 8466625

[13] Belperio JA Keane MP Arenberg DA Addison CL Ehlert JE Burdick MD CXC chemokines in angiogenesis. J Leukoc Biol (2000) 68(1):1–8 10914483

[14] Strieter RM Burdick MD Gomperts BN Belperio JA Keane MP CXC chemokines in angiogenesis. Cytokine Growth Factor Rev (2005) 16(6):593–609 10.1016/j.cytogfr.2005.04.007 16046180

[15] Kuziel WA Morgan SJ Dawson TC Griffin S Smithies O Ley K Severe reduction in leukocyte adhesion and monocyte extravasation in mice deficient in CC chemokine receptor 2. Proc Natl Acad Sci U S A (1998) 94:12053–8 10.1073/pnas.94.22.12053 9342361 PMC23699

[16] Reichel CA Puhr-Westerheide D Zuchtriegel G Uhl B Berberich N Zahler S C-C motif chemokine CCL3 and canonical neutrophil attractants promote neutrophil extravasation through common and distinct mechanisms. Blood (2012) 120(4):880–90 10.1182/blood-2012-01-402164 22674804

[17] Sanchez-Sanchez N Riol-Blanco L de la Rosa G Puig-Kroger A Garcia-Bordas J Martin D Chemokine receptor CCR7 induces intracellular signaling that inhibits apoptosis of mature dendritic cells. Blood (2004) 104(3):619–25 10.1182/blood-2003-11-3943 15059845

[18] Wright LM Maloney W Yu X Kindle L Collin-Osdoby P Osdoby P Stromal cell-derived factor-1 binding to its chemokine receptor CXCR4 on precursor cells promotes the chemotactic recruitment, development and survival of human osteoclasts. Bone (2005) 36(5):840–53 10.1016/j.bone.2005.01.021 15794931

[19] Gulick RM Lalezari J Goodrich J Clumeck N DeJesus E Horban A Maraviroc for previously treated patients with R5 HIV-1 infection. N Engl J Med (2008) 359(14):1429–41 10.1056/NEJMoa0803152 18832244 PMC3078519

[20] Hardy WD Gulick RM Mayer H Fatkenheuer G Nelson M Heera J Two-year safety and virologic efficacy of maraviroc in treatment-experienced patients with CCR5-tropic HIV-1 infection: 96-week combined analysis of MOTIVATE 1 and 2. J Acquir Immune Defic Syndr (2010) 55(5):558–64 10.1097/QAI.0b013e3181ee3d82 20703158 PMC3321258

[21] DiPersio JF Micallef IN Stiff PJ Bolwell BJ Maziarz RT Jacobsen E Phase III prospective randomized double-blind placebo-controlled trial of plerixafor plus granulocyte colony-stimulating factor compared with placebo plus granulocyte colony-stimulating factor for autologous stem-cell mobilization and transplantation for patients with non-Hodgkin’s lymphoma. J Clin Oncol (2009) 27(28):4767–73 10.1200/JCO.2008.20.7209 19720922

[22] Brave M Farrell A Ching LS Ocheltree T Pope MS Lee SL FDA review summary: mozobil in combination with granulocyte colony-stimulating factor to mobilize hematopoietic stem cells to the peripheral blood for collection and subsequent autologous transplantation. Oncology (2010) 78(3–4):282–8 10.1159/000315736 20530974

[23] Wijtmans M Verzijl D Leurs R de Esch IJ Smit MJ Towards small-molecule CXCR3 ligands with clinical potential. ChemMedChem (2008) 3(6):861–72 10.1002/cmdc.200700365 18442035

[24] Allegretti M Cesta MC Garin A Proudfoot AE Current status of chemokine receptor inhibitors in development. Immunol Lett (2012) 145(1–2):68–78 10.1016/j.imlet.2012.04.003 22698186

[25] Schall TJ Proudfoot AE Overcoming hurdles in developing successful drugs targeting chemokine receptors. Nat Rev Immunol (2011) 11(5):355–63 10.1038/nri2972 21494268

[26] Ma Q Jones D Borghesani PR Segal RA Nagasawa T Kishimoto T Impaired B-lymphopoiesis, myelopoiesis, and derailed cerebellar neuron migration in CXCR4- and SDF-1 deficient mice. Proc Natl Acad Sci U S A (1998) 95:9448–53 10.1073/pnas.95.16.9448 9689100 PMC21358

[27] Tachibana K Hirota S Iizasa H Yoshida H Kawabata K Kataoka Y The chemokine receptor CXCR4 is essential for vascularization of the gastrointestinal tract. Nature (1998) 393(6685):591–4 10.1038/31261 9634237

[28] Roth BL Chuang DM Multiple mechanisms of serotonergic signal transduction. Life Sci (1987) 41(9):1051–64 10.1016/0024-3205(87)90621-7 2441225

[29] Kenakin T Agonist-receptor efficacy. II. Agonist trafficking of receptor signals. Trends Pharmacol Sci (1995) 16(7):232–8 10.1016/S0165-6147(00)89032-X 7667897

[30] Kahsai AW Xiao K Rajagopal S Ahn S Shukla AK Sun J Multiple ligand-specific conformations of the beta2-adrenergic receptor. Nat Chem Biol (2011) 7(10):692–700 10.1038/nchembio.634 21857662 PMC3404607

[31] Liu JJ Horst R Katritch V Stevens RC Wuthrich K Biased signaling pathways in beta2-adrenergic receptor characterized by 19F-NMR. Science (2012) 335(6072):1106–10 10.1126/science.1215802 22267580 PMC3292700

[32] Mary S Damian M Louet M Floquet N Fehrentz JA Marie J Ligands and signaling proteins govern the conformational landscape explored by a G protein-coupled receptor. Proc Natl Acad Sci U S A (2012) 109(21):8304–9 10.1073/pnas.1119881109 22573814 PMC3361445

[33] Wootten D Simms J Miller LJ Christopoulos A Sexton PM Polar transmembrane interactions drive formation of ligand-specific and signal pathway-biased family B G protein-coupled receptor conformations. Proc Natl Acad Sci U S A (2013) 110(13):5211–6 10.1073/pnas.1221585110 23479653 PMC3612682

[34] Rosenkilde MM McLean KA Holst PJ Schwartz TW The CXC chemokine receptor encoded by herpesvirus saimiri, ECRF3, shows ligand-regulated signaling through Gi, Gq, and G12/13 proteins but constitutive signaling only through Gi and G12/13 proteins. J Biol Chem (2004) 279(31):32524–33 10.1074/jbc.M313392200 15155729

[35] Forster R Davalos-Misslitz AC Rot A CCR7 and its ligands: balancing immunity and tolerance. Nat Rev Immunol (2008) 8(5):362–71 10.1038/nri2297 18379575

[36] Patel DD Koopmann W Imai T Whichard LP Yoshie O Krangel MS Chemokines have diverse abilities to form solid phase gradients. Clin Immunol (2001) 99(1):43–52 10.1006/clim.2000.4997 11286540

[37] de Paz JL Moseman EA Noti C Polito L Von Andrian UH Seeberger PH Profiling heparin-chemokine interactions using synthetic tools. ACS Chem Biol (2007) 2(11):735–44 10.1021/cb700159m 18030990 PMC2716178

[38] Bardi G Lipp M Baggiolini M Loetscher P The T cell chemokine receptor CCR7 is internalized on stimulation with ELC, but not with SLC. Eur J Immunol (2001) 31(11):3291–7 10.1002/1521-4141(200111)31:11<3291::AID-IMMU3291>3.0.CO;2-Z 11745346

[39] Byers MA Calloway PA Shannon L Cunningham HD Smith S Li F Arrestin 3 mediates endocytosis of CCR7 following ligation of CCL19 but not CCL21. J Immunol (2008) 181(7):4723–32 10.4049/jimmunol.181.7.4723 18802075 PMC7877961

[40] Zidar DA Violin JD Whalen EJ Lefkowitz RJ Selective engagement of G protein coupled receptor kinases (GRKs) encodes distinct functions of biased ligands. Proc Natl Acad Sci U S A (2009) 106(24):9649–54 10.1073/pnas.0904361106 19497875 PMC2689814

[41] Ricart BG John B Lee D Hunter CA Hammer DA Dendritic cells distinguish individual chemokine signals through CCR7 and CXCR4. J Immunol (2011) 186(1):53–61 10.4049/jimmunol.1002358 21106854

[42] Kledal TN Rosenkilde MM Schwartz TW Selective recognition of the membrane-bound CX3C chemokine, fractalkine, by the human cytomegalovirus-encoded broad-spectrum receptor US28. FEBS Lett (1998) 441(2):209–14 10.1016/S0014-5793(98)01551-8 9883886

[43] Gao JL Murphy PM Human cytomegalovirus open reading frame US28 encodes a functional beta chemokine receptor. J Biol Chem (1994) 269(46):28539–42 7961796

[44] Kuhn DE Beall CJ Kolattukudy PE The cytomegalovirus US28 protein binds multiple CC chemokines with high affinity. Biochem Biophys Res Commun (1995) 211(1):325–30 10.1006/bbrc.1995.1814 7540006

[45] Mizoue LS Sullivan SK King DS Kledal TN Schwartz TW Bacon KB Molecular determinants of receptor binding and signaling by the CX3C chemokine fractalkine. J Biol Chem (2001) 276(36):33906–14 10.1074/jbc.M101348200 11432858

[46] Casarosa P Bakker RA Verzijl D Navis M Timmerman H Leurs R Constitutive signaling of the human cytomegalovirus-encoded chemokine receptor US28. J Biol Chem (2001) 276(2):1133–7 10.1074/jbc.M008965200 11050102

[47] Fraile-Ramos A Kohout TA Waldhoer M Marsh M Endocytosis of the viral chemokine receptor US28 does not require beta-arrestins but is dependent on the clathrin-mediated pathway. Traffic (2003) 4(4):243–53 10.1034/j.1600-0854.2003.00079.x 12694563

[48] McLean KA Holst PJ Martini L Schwartz TW Rosenkilde MM Similar activation of signal transduction pathways by the herpesvirus-encoded chemokine receptors US28 and ORF74. Virology (2004) 325(2):241–51 10.1016/j.virol.2004.04.027 15246264

[49] Casarosa P Waldhoer M LiWang PJ Vischer HF Kledal T Timmerman H CC and CX3C chemokines differentially interact with the N terminus of the human cytomegalovirus-encoded US28 receptor. J Biol Chem (2005) 280(5):3275–85 10.1074/jbc.M407536200 15546882

[50] Ahuja SK Murphy PM Molecular piracy of mammalian interleukin-8 receptor type B by herpesvirus saimiri. J Biol Chem (1993) 268(28):20691–4 8407886

[51] Arvanitakis L Geras-Raaka E Varma A Gershengorn MC Cesarman E Human herpesvirus KSHV encodes a constitutively active G-protein-coupled receptor linked to cell proliferation. Nature (1997) 385(6614):347–50 10.1038/385347a0 9002520

[52] Geras-Raaka E Varma A Ho H Clark-Lewis I Gershengorn MC Human interferon-gamma-inducible protein 10 (IP-10) inhibits constitutive signaling of Kaposi’s sarcoma-associated herpesvirus G protein-coupled receptor. J Exp Med (1998) 188(2):405–8 10.1084/jem.188.2.405 9670053 PMC2212452

[53] Geras-Raaka E Varma A Clark-Lewis I Gershengorn MC Kaposi’s sarcoma-associated herpesvirus (KSHV) chemokine vMIP-II and human SDF-1alpha inhibit signaling by KSHV G protein-coupled receptor. Biochem Biophys Res Commun (1998) 253(3):725–7 10.1006/bbrc.1998.9557 9918794

[54] Rosenkilde MM Kledal TN Brauner-Osborne H Schwartz TW Agonists and inverse agonists for the herpesvirus 8-encoded constitutively active seven-transmembrane oncogene product, ORF-74. J Biol Chem (1999) 274(2):956–61 10.1074/jbc.274.2.956 9873037

[55] Rosenkilde MM Schwartz TW Potency of ligands correlates with affinity measured against agonist and inverse agonists but not against neutral ligand in constitutively active chemokine receptor. Mol Pharmacol (2000) 57(3):602–9 10692502 10.1124/mol.57.3.602

[56] Rajagopal S Bassoni DL Campbell JJ Gerard NP Gerard C Wehrman TS Biased agonism as a mechanism for differential signaling by chemokine receptors. J Biol Chem (2013) 288(49):35039–48 10.1074/jbc.M113.479113 24145037 PMC3853256

[57] Sauty A Colvin RA Wagner L Rochat S Spertini F Luster AD CXCR3 internalization following T cell-endothelial cell contact: preferential role of IFN-inducible T cell alpha chemoattractant (CXCL11). J Immunol (2001) 167(12):7084–93 10.4049/jimmunol.167.12.7084 11739530

[58] Feniger-Barish R Ran M Zaslaver A Ben Baruch A Differential modes of regulation of cxc chemokine-induced internalization and recycling of human CXCR1 and CXCR2. Cytokine (1999) 11(12):996–1009 10.1006/cyto.1999.0510 10623425

[59] Ahuja SK Lee JM Murphy PM CXC chemokines bind to unique sets of selectivity determinants that can function independently and are broadly distributed on multiple domains of human interleukin-8 receptor B. J Biol Chem (1996) 271(1):225–32 10.1074/jbc.271.1.225 8550564

[60] Colvin RA Campanella GS Sun J Luster AD Intracellular domains of CXCR3 that mediate CXCL9, CXCL10, and CXCL11 function. J Biol Chem (2004) 279(29):30219–27 10.1074/jbc.M403595200 15150261

[61] Rosenkilde MM Andersen MB Nygaard R Frimurer TM Schwartz TW Activation of the CXCR3 chemokine receptor through anchoring of a small molecule chelator ligand between TM-III, -IV, and -VI. Mol Pharmacol (2007) 71(3):930–41 10.1124/mol.106.030031 17170198

[62] Rosenkilde MM Gerlach LO Jakobsen JS Skerlj RT Bridger GJ Schwartz TW Molecular mechanism of AMD3100 antagonism in the CXCR4 receptor: transfer of binding site to the CXCR3 receptor. J Biol Chem (2004) 279(4):3033–41 10.1074/jbc.M309546200 14585837

[63] Zohar Y Wildbaum G Novak R Salzman AL Thelen M Alon R CXCL11-dependent induction of FOXP3-negative regulatory T cells suppresses autoimmune encephalomyelitis. J Clin Invest (2014) 124(5):2009–22 10.1172/JCI71951 24713654 PMC4001543

[64] Savino B Borroni EM Torres NM Proost P Struyf S Mortier A Recognition versus adaptive up-regulation and degradation of CC chemokines by the chemokine decoy receptor D6 are determined by their N-terminal sequence. J Biol Chem (2009) 284(38):26207–15 10.1074/jbc.M109.029249 19632987 PMC2758019

[65] Borroni EM Cancellieri C Vacchini A Benureau Y Lagane B Bachelerie F Beta-arrestin-dependent activation of the cofilin pathway is required for the scavenging activity of the atypical chemokine receptor D6. Sci Signal (2013) 6(273):ra30–3 10.1126/scisignal.2003627 23633677

[66] Monteclaro FS Charo IF The amino-terminal extracellular domain of the MCP-1 receptor, but not the RANTES/MIP-1alpha receptor, confers chemokine selectivity. Evidence for a two-step mechanism for MCP-1 receptor activation. J Biol Chem (1996) 271(32):19084–92 10.1074/jbc.271.32.19084 8702581

[67] Monteclaro FS Charo IF The amino-terminal domain of CCR2 is both necessary and sufficient for high affinity binding of monocyte chemoattractant protein 1. J Biol Chem (1997) 272(37):23186–90 10.1074/jbc.272.37.23186 9287323

[68] Allen SJ Crown SE Handel TM Chemokine: receptor structure, interactions, and antagonism. Annu Rev Immunol (2007) 25:787–820 10.1146/annurev.immunol.24.021605.090529 17291188

[69] Mortier A Van DJ Proost P Overview of the mechanisms regulating chemokine activity and availability. Immunol Lett (2012) 145(1–2):2–9 10.1016/j.imlet.2012.04.015 22698177

[70] Drury LJ Ziarek JJ Gravel S Veldkamp CT Takekoshi T Hwang ST Monomeric and dimeric CXCL12 inhibit metastasis through distinct CXCR4 interactions and signaling pathways. Proc Natl Acad Sci U S A (2011) 108(43):17655–60 10.1073/pnas.1101133108 21990345 PMC3203819

[71] Covic L Gresser AL Talavera J Swift S Kuliopulos A Activation and inhibition of G protein-coupled receptors by cell-penetrating membrane-tethered peptides. Proc Natl Acad Sci U S A (2002) 99(2):643–8 10.1073/pnas.022460899 11805322 PMC117359

[72] Quoyer J Janz JM Luo J Ren Y Armando S Lukashova V Pepducin targeting the C-X-C chemokine receptor type 4 acts as a biased agonist favoring activation of the inhibitory G protein. Proc Natl Acad Sci U S A (2013) 110(52):E5088–97 10.1073/pnas.1312515110 24309376 PMC3876208

[73] Bleul CC Farzan M Choe H Parolin C Clark-Lewis I Sodroski J The lymphocyte chemoattractant SDF-1 is a ligand for LESTR/fusin and blocks HIV-1 entry. Nature (1996) 382(6594):829–33 10.1038/382829a0 8752280

[74] Cheng ZJ Zhao J Sun Y Hu W Wu YL Cen B Beta-arrestin differentially regulates the chemokine receptor CXCR4-mediated signaling and receptor internalization, and this implicates multiple interaction sites between beta-arrestin and CXCR4. J Biol Chem (2000) 275(4):2479–85 10.1074/jbc.275.4.2479 10644702

[75] Sun Y Cheng Z Ma L Pei G Beta-arrestin2 is critically involved in CXCR4-mediated chemotaxis, and this is mediated by its enhancement of p38 MAPK activation. J Biol Chem (2002) 277(51):49212–9 10.1074/jbc.M207294200 12370187

[76] Balabanian K Lagane B Infantino S Chow KY Harriague J Moepps B The chemokine SDF-1/CXCL12 binds to and signals through the orphan receptor RDC1 in T lymphocytes. J Biol Chem (2005) 280(42):35760–6 10.1074/jbc.M508234200 16107333

[77] Burns JM Summers BC Wang Y Melikian A Berahovich R Miao Z A novel chemokine receptor for SDF-1 and I-TAC involved in cell survival, cell adhesion, and tumor development. J Exp Med (2006) 203(9):2201–13 10.1084/jem.20052144 16940167 PMC2118398

[78] Boldajipour B Mahabaleshwar H Kardash E Reichman-Fried M Blaser H Minina S Control of chemokine-guided cell migration by ligand sequestration. Cell (2008) 132(3):463–73 10.1016/j.cell.2007.12.034 18267076

[79] Rajagopal S Kim J Ahn S Craig S Lam CM Gerard NP Beta-arrestin- but not G protein-mediated signaling by the “decoy” receptor CXCR7. Proc Natl Acad Sci U S A (2010) 107(2):628–32 10.1073/pnas.0912852107 20018651 PMC2818968

[80] Bonecchi R Locati M Galliera E Vulcano M Sironi M Fra AM Differential recognition and scavenging of native and truncated macrophage-derived chemokine (macrophage-derived chemokine/CC chemokine ligand 22) by the D6 decoy receptor. J Immunol (2004) 172(8):4972–6 10.4049/jimmunol.172.8.4972 15067078

[81] Loetscher P Pellegrino A Gong JH Mattioli I Loetscher M Bardi G The ligands of CXC chemokine receptor 3, I-TAC, Mig, and IP10, are natural antagonists for CCR3. J Biol Chem (2001) 276(5):2986–91 10.1074/jbc.M005652200 11110785

[82] Petkovic V Moghini C Paoletti S Uguccioni M Gerber B Eotaxin-3/CCL26 is a natural antagonist for CC chemokine receptors 1 and 5. A human chemokine with a regulatory role. J Biol Chem (2004) 279(22):23357–63 10.1074/jbc.M309283200 15039444

[83] Nibbs RJ Salcedo TW Campbell JD Yao XT Li Y Nardelli B C-C chemokine receptor 3 antagonism by the beta-chemokine macrophage inflammatory protein 4, a property strongly enhanced by an amino-terminal alanine-methionine swap. J Immunol (2000) 164(3):1488–97 10.4049/jimmunol.164.3.1488 10640766

[84] Blanpain C Migeotte I Lee B Vakili J Doranz BJ Govaerts C CCR5 binds multiple CC-chemokines: MCP-3 acts as a natural antagonist. Blood (1999) 94(6):1899–905 10477718

[85] Chou CC Fine JS Pugliese-Sivo C Gonsiorek W Davies L Deno G Pharmacological characterization of the chemokine receptor, hCCR1 in a stable transfectant and differentiated HL-60 cells: antagonism of hCCR1 activation by MIP-1beta. Br J Pharmacol (2002) 137(5):663–75 10.1038/sj.bjp.0704907 12381680 PMC1573530

[86] Ogilvie P Bardi G Clark-Lewis I Baggiolini M Uguccioni M Eotaxin is a natural antagonist for CCR2 and an agonist for CCR5. Blood (2001) 97(7):1920–4 10.1182/blood.V97.7.1920 11264152

[87] Ogilvie P Paoletti S Clark-Lewis I Uguccioni M Eotaxin-3 is a natural antagonist for CCR2 and exerts a repulsive effect on human monocytes. Blood (2003) 102(3):789–94 10.1182/blood-2002-09-2773 12689946

[88] Ogilvie P Thelen S Moepps B Gierschik P da Silva Campos AC Baggiolini M Unusual chemokine receptor antagonism involving a mitogen-activated protein kinase pathway. J Immunol (2004) 172(11):6715–22 10.4049/jimmunol.172.11.6715 15153488

[89] Imai T Hieshima K Haskell C Baba M Nagira M Nishimura M Identification and molecular characterization of fractalkine receptor CX3CR1, which mediates both leukocyte migration and adhesion. Cell (1997) 91(4):521–30 10.1016/S0092-8674(00)80438-9 9390561

[90] Loetscher M Berber B Loetscher P Jones SA Piali L Clark-Lewis I Chemokine receptor specific for IP10 and mig: structure, function, and expression in activated T-lymphocytes. J Exp Med (1996) 184:963–9 10.1084/jem.184.3.963 9064356 PMC2192763

[91] Oberlin E Amara A Bachelerie F Bessia C Virelizier JL Renzana-Seisdedos F The CXC chemokine SDF-1 is the ligand for LESTR/fusin and prevents infection by T-cell-line-adapted HIV-1. Nature (1996) 382(6594):833–5 10.1038/382833a0 8752281

[92] Van Damme J Wuyts A Froyen G Van Collie E Struyf S Billiau A Granulocyte chemotactic protein-2 and related CXC chemokines: from gene regulation to receptor usage. J Leukoc Biol (1997) 62(5):563–9 9365109 10.1002/jlb.62.5.563

[93] Gershengorn MC Geras-Raaka E Varma A Clark-Lewis I Chemokines activate Kaposi’s sarcoma-associated herpesvirus G protein-coupled receptor in mammalian cells in culture. J Clin Invest (1998) 102(8):1469–72 10.1172/JCI4461 9788958 PMC508995

[94] Rosenkilde MM Kledal TN Holst PJ Schwartz TW Selective elimination of high constitutive activity or chemokine binding in the human herpesvirus 8 encoded seven transmembrane oncogene ORF74. J Biol Chem (2000) 275(34):26309–15 10.1074/jbc.M003800200 10842179

[95] Kenakin TP Ambrose JR Irving PE The relative efficiency of beta adrenoceptor coupling to myocardial inotropy and diastolic relaxation: organ-selective treatment for diastolic dysfunction. J Pharmacol Exp Ther (1991) 257(3):1189–97 1675290

[96] Kenakin T The potential for selective pharmacological therapies through biased receptor signaling. BMC Pharmacol Toxicol (2012) 13:3 10.1186/2050-6511-13-3 22947056 PMC3506267

[97] Gunn MD Chemokine mediated control of dendritic cell migration and function. Semin Immunol (2003) 15(5):271–6 10.1016/j.smim.2003.08.004 15001176

[98] Carlsen HS Haraldsen G Brandtzaeg P Baekkevold ES Disparate lymphoid chemokine expression in mice and men: no evidence of CCL21 synthesis by human high endothelial venules. Blood (2005) 106(2):444–6 10.1182/blood-2004-11-4353 15863780

[99] Randolph GJ Angeli V Swartz MA Dendritic-cell trafficking to lymph nodes through lymphatic vessels. Nat Rev Immunol (2005) 5(8):617–28 10.1038/nri1670 16056255

[100] Nandagopal S Wu D Lin F Combinatorial guidance by CCR7 ligands for T lymphocytes migration in co-existing chemokine fields. PLoS One (2011) 6(3):e18183 10.1371/journal.pone.0018183 21464944 PMC3064588

[101] Bridger GJ Skerlj RT Padmanabhan S Martellucci SA Henson GW Struyf S Synthesis and structure-activity relationships of phenylenebis(methylene)-linked bis-azamacrocycles that inhibit HIV-1 and HIV-2 replication by antagonism of the chemokine receptor CXCR4. J Med Chem (1999) 42(19):3971–81 10.1021/jm990211i 10508445

[102] Gerlach LO Skerlj RT Bridger GJ Schwartz TW Molecular interactions of cyclam and bicyclam non-peptide antagonists with the CXCR4 chemokine receptor. J Biol Chem (2001) 276(17):14153–60 10.1074/jbc.M010429200 11154697

[103] Gerlach LO Jakobsen JS Jensen KP Rosenkilde MR Skerlj RT Ryde U Metal ion enhanced binding of AMD3100 to Asp262 in the CXCR4 receptor. Biochemistry (2003) 42(3):710–7 10.1021/bi0264770 12534283

[104] Hatse S Princen K Vermeire K Gerlach LO Rosenkilde MM Schwartz TW Mutations at the CXCR4 interaction sites for AMD3100 influence anti-CXCR4 antibody binding and HIV-1 entry. FEBS Lett (2003) 546(2–3):300–6 10.1016/S0014-5793(03)00609-4 12832058

[105] Rosenkilde MM Gerlach LO Hatse S Skerlj RT Schols D Bridger GJ Molecular mechanism of action of monocyclam versus bicyclam non-peptide antagonists in the CXCR4 chemokine receptor. J Biol Chem (2007) 282(37):27354–65 10.1074/jbc.M704739200 17599916

[106] Zhang WB Navenot JM Haribabu B Tamamura H Hiramatu K Omagari A A point mutation that confers constitutive activity to CXCR4 reveals that T140 is an inverse agonist and that AMD3100 and ALX40-4C are weak partial agonists. J Biol Chem (2002) 277(27):24515–21 10.1074/jbc.M200889200 11923301

[107] Manglik A Kobilka B The role of protein dynamics in GPCR function: insights from the betaAR and rhodopsin. Curr Opin Cell Biol (2014) 27C:136–43 10.1016/j.ceb.2014.01.008 24534489 PMC3986065

[108] Schwartz TW Frimurer TM Holst B Rosenkilde MM Elling CE Molecular mechanism of 7TM receptor activation – a global toggle switch model. Annu Rev Pharmacol Toxicol (2006) 46:481–519 10.1146/annurev.pharmtox.46.120604.141218 16402913

[109] Rosenkilde MM Benned-Jensen T Frimurer TM Schwartz TW The minor binding pocket: a major player in 7TM receptor activation. Trends Pharmacol Sci (2010) 31(12):567–74 10.1016/j.tips.2010.08.006 20870300

[110] Katritch V Cherezov V Stevens RC Structure-function of the g protein-coupled receptor superfamily. Annu Rev Pharmacol Toxicol (2013) 53:531–56 10.1146/annurev-pharmtox-032112-135923 23140243 PMC3540149

[111] Nygaard R Frimurer TM Holst B Rosenkilde MM Schwartz TW Ligand binding and micro-switches in 7TM receptor structures. Trends Pharmacol Sci (2009) 30(5):249–59 10.1016/j.tips.2009.02.006 19375807

[112] Venkatakrishnan AJ Deupi X Lebon G Tate CG Schertler GF Babu MM Molecular signatures of G-protein-coupled receptors. Nature (2013) 494(7436):185–94 10.1038/nature11896 23407534

[113] Wu B Chien EY Mol CD Fenalti G Liu W Katritch V Structures of the CXCR4 chemokine GPCR with small-molecule and cyclic peptide antagonists. Science (2010) 330(6007):1066–76 10.1126/science.1194396 20929726 PMC3074590

[114] Tan Q Zhu Y Li J Chen Z Han GW Kufareva I Structure of the CCR5 chemokine receptor-HIV entry inhibitor maraviroc complex. Science (2013) 341(6152):1387–90 10.1126/science.1241475 24030490 PMC3819204

[115] Park SH Das BB Casagrande F Tian Y Nothnagel HJ Chu M Structure of the chemokine receptor CXCR1 in phospholipid bilayers. Nature (2012) 491(7426):779–83 10.1038/nature11580 23086146 PMC3700570

[116] Rasmussen SG DeVree BT Zou Y Kruse AC Chung KY Kobilka TS Crystal structure of the beta2 adrenergic receptor-Gs protein complex. Nature (2011) 477(7366):549–55 10.1038/nature10361 21772288 PMC3184188

[117] Chung KY Rasmussen SG Liu T Li S DeVree BT Chae PS Conformational changes in the G protein Gs induced by the beta2 adrenergic receptor. Nature (2011) 477(7366):611–5 10.1038/nature10488 21956331 PMC3448949

[118] Shukla AK Manglik A Kruse AC Xiao K Reis RI Tseng WC Structure of active beta-arrestin-1 bound to a G-protein-coupled receptor phosphopeptide. Nature (2013) 497(7447):137–41 10.1038/nature12120 23604254 PMC3654799

[119] Warne T Edwards PC Leslie AG Tate CG Crystal structures of a stabilized beta1-adrenoceptor bound to the biased agonists bucindolol and carvedilol. Structure (2012) 20(5):841–9 10.1016/j.str.2012.03.014 22579251 PMC3384003

[120] Wacker D Wang C Katritch V Han GW Huang XP Vardy E Structural features for functional selectivity at serotonin receptors. Science (2013) 340(6132):615–9 10.1126/science.1232808 23519215 PMC3644390

[121] Rasmussen SG Choi HJ Fung JJ Pardon E Casarosa P Chae PS Structure of a nanobody-stabilized active state of the beta(2) adrenoceptor. Nature (2011) 469(7329):175–80 10.1038/nature09648 21228869 PMC3058308

[122] Warne T Moukhametzianov R Baker JG Nehme R Edwards PC Leslie AG The structural basis for agonist and partial agonist action on a beta(1)-adrenergic receptor. Nature (2011) 469(7329):241–4 10.1038/nature09746 21228877 PMC3023143

[123] Fenalti G Giguere PM Katritch V Huang XP Thompson AA Cherezov V Molecular control of delta-opioid receptor signalling. Nature (2014) 506(7487):191–6 10.1038/nature12944 24413399 PMC3931418

[124] Schwartz TW Locating ligand-binding sites in 7TM receptors by protein engineering. Curr Opin Biotechnol (1994) 5:434–44 10.1016/0958-1669(94)90054-X 7765177

[125] Ballesteros JA Weinstein H Integrated methods for the construction of three-dimensional models and computational probing of structure-function relations in G protein-coupled receptors. In: Sealfon SC editor. Receptor Molecular Biology. San Diego: Academic press (1995). p. 366–428

[126] Lagane B Ballet S Planchenault T Balabanian K Le PE Blanpain C Mutation of the DRY motif reveals different structural requirements for the CC chemokine receptor 5-mediated signaling and receptor endocytosis. Mol Pharmacol (2005) 67(6):1966–76 10.1124/mol.104.009779 15761117

[127] Hoffmann F Muller W Schutz D Penfold ME Wong YH Schulz S Rapid uptake and degradation of CXCL12 depend on CXCR7 carboxyl-terminal serine/threonine residues. J Biol Chem (2012) 287(34):28362–77 10.1074/jbc.M111.335679 22736769 PMC3436560

[128] Yoshida T Imai T Kakizaki M Nishimura M Takagi S Yoshie O Identification of single C motif-1/lymphotactin receptor XCR1. J Biol Chem (1998) 273(26):16551–4 10.1074/jbc.273.26.16551 9632725

[129] Chandrasekar B Bysani S Mummidi S CXCL16 signals via Gi, phosphatidylinositol 3-kinase, Akt, I kappa B kinase, and nuclear factor-kappa B and induces cell-cell adhesion and aortic smooth muscle cell proliferation. J Biol Chem (2004) 279(5):3188–96 10.1074/jbc.M311660200 14625285

[130] Rosenkilde MM Kledal TN Schwartz TW High constitutive activity of a virus-encoded 7TM receptor in the absence of the conserved DRY-motif (Asp-Arg-Tyr) in transmembrane helix 3. Mol Pharmacol (2005) 68:11–9 10.1124/mol.105.011239 15788740

[131] Flanagan CA A GPCR that is not “DRY”. Mol Pharmacol (2005) 68(1):1–3 10.1124/mol.105.014183 15855406

[132] Valentin-Hansen L Groenen M Nygaard R Frimurer TM Holliday ND Schwartz TW The arginine of the DRY motif in transmembrane segment III functions as a balancing micro-switch in the activation of the beta2-adrenergic receptor. J Biol Chem (2012) 287(38):31973–82 10.1074/jbc.M112.348565 22843684 PMC3442529

[133] Steen A Thiele S Guo D Hansen LS Frimurer TM Rosenkilde MM Biased and constitutive signaling in the CC-chemokine receptor CCR5 by manipulating the interface between transmembrane helices 6 and 7. J Biol Chem (2013) 288(18):12511–21 10.1074/jbc.M112.449587 23493400 PMC3642299

[134] Steen A Sparre-Ulrich AH Thiele S Guo D Frimurer TM Rosenkilde MM Gating function of isoleucine-116 in TM-3 (position III:16/3.40) for the activity state of the CC-chemokine receptor 5 (CCR5). Br J Pharmacol (2014) 171(6):1566–79 10.1111/bph.12553 24328926 PMC3954493

[135] Valentin-Hansen L Holst B Frimurer TM Schwartz TW PheVI:09 (Phe6.44) as a sliding microswitch in seven-transmembrane (7TM) G protein-coupled receptor activation. J Biol Chem (2012) 287(52):43516–26 10.1074/jbc.M112.395137 23135271 PMC3527938

[136] Jensen PC Thiele S Ulven T Schwartz TW Rosenkilde MM Positive versus negative modulation of different endogenous chemokines for CC-chemokine receptor 1 by small molecule agonists through allosteric versus orthosteric binding. J Biol Chem (2008) 283(34):23121–8 10.1074/jbc.M803458200 18559339

[137] Thiele S Malmgaard-Clausen M Engel-Andreasen J Steen A Rummel PC Nielsen MC Modulation in selectivity and allosteric properties of small-molecule ligands for CC-chemokine receptors. J Med Chem (2012) 55(18):8164–77 10.1021/jm301121j 22957890

[138] Chalikiopoulos A Thiele S Malmgaard-Clausen M Rydberg P Isberg V Ulven T Structure-activity relationships and identification of optimized CC-chemokine receptor CCR1, 5, and 8 metal-ion chelators. J Chem Inf Model (2013) 53(11):2863–73 10.1021/ci4003848 24083637

[139] Rummel PC Thiele S Hansen LS Petersen TP Sparre-Ulrich AH Ulven T Extracellular disulfide bridges serve different purposes in two homologous chemokine receptors, CCR1 and CCR5. Mol Pharmacol (2013) 84(3):335–45 10.1124/mol.113.086702 23765404

[140] Wisler JW DeWire SM Whalen EJ Violin JD Drake MT Ahn S A unique mechanism of beta-blocker action: carvedilol stimulates beta-arrestin signaling. Proc Natl Acad Sci U S A (2007) 104(42):16657–62 10.1073/pnas.0707936104 17925438 PMC2034221

[141] Kim IM Tilley DG Chen J Salazar NC Whalen EJ Violin JD Beta-blockers alprenolol and carvedilol stimulate beta-arrestin-mediated EGFR transactivation. Proc Natl Acad Sci U S A (2008) 105(38):14555–60 10.1073/pnas.0804745105 18787115 PMC2567217

[142] Noma T Lemaire A Naga Prasad SV Barki-Harrington L Tilley DG Chen J Beta-arrestin-mediated beta1-adrenergic receptor transactivation of the EGFR confers cardioprotection. J Clin Invest (2007) 117(9):2445–58 10.1172/JCI31901 17786238 PMC1952636

[143] Bristow MR Beta-adrenergic receptor blockade in chronic heart failure. Circulation (2000) 101(5):558–69 10.1161/01.CIR.101.5.558 10662755

[144] Scholten DJ Canals M Maussang D Roumen L Smit MJ Wijtmans M Pharmacological modulation of chemokine receptor function. Br J Pharmacol (2012) 165(6):1617–43 10.1111/j.1476-5381.2011.01551.x 21699506 PMC3372818

